# Clinical validation and experiences of the microfluidics sperm selection device ZyMōt™ for standard IVF

**DOI:** 10.5935/1518-0557.20240104

**Published:** 2025

**Authors:** Emma Adolfsson, Johanna Ingberg, Emma Igersten, Therese Bohlin

**Affiliations:** 1 Department of Obstetrics and Gynecology, Faculty of Medicine and Health, Örebro University, Örebro, Sweden; 2 School of Health Science, Örebro University, Örebro, Sweden

**Keywords:** microfluidics, sperm selection device, density gradient centrifugation, sperm selection, *in vitro* fertilization, clinical validation

## Abstract

**Objective:**

Clinical validation of sperm selection device ZyMōt™ for standard IVF.

**Methods:**

The pre-clinical validation of ZyMōt™ included several steps. First, split semen preparation compared density gradient centrifugation (DGC) to ZyMōt™ with primary outcome fraction and absolute number of progressive motile sperm. Second, sibling oocytes were fertilized with sperms prepared with DGC and sperms selected by ZyMōt™, primary endpoint fertilization rate, utility rate, embryo development pace quality. After this, DGC was replaced by ZyMōt™, first without centrifugation steps, and then with a five-minute centrifugation step and subsequent media change prior to gamete co-incubation. Endpoint was assessment of key performance indicators against previous results using DGC for standard IVF.

**Results:**

ZyMōt™ resulted in purer sperm selection compared to DGC (fraction progressive motile sperm 97.2±3.1% vs. 83.0±14.1%, p<0.01). Fertilization of sibling oocytes resulted in similar fertilization rates and utility rates, and no differences in embryo development pace or quality. However, after changing sperm selection protocol from DGC to ZyMōt™ for standard IVF for all fresh semen samples with motile sperm, the fertilization rates and utility rates were significantly reduced, and cases of total failure of fertilization increased substantially. Adding five-minute centrifugation and media change after centrifugation to the sperm selection protocol restored fertilization rate, including total failure of fertilization rate, to normal.

**Conclusions:**

To conclude, the ZyMōt™ sperm selection device is suitable for standard IVF only after inclusion of five minutes centrifugation and subsequent media change prior to gamete co-incubation.

## INTRODUCTION

The ESHRE revised guidelines for good laboratory practice ( ESHRE Guideline Group on Good Practice in IVF Labs, 2016 ) states that sperm selection prior to assisted reproduction aims to eliminate seminal plasma, debris and contaminants, to concentrate progressively motile sperm and select against morphologically abnormal sperm. The two most common and accepted methods are the swim-up technique and density gradient centrifugation (DGC).

Swim-up techniques are based on motility. Culture medium is layered over the liquefied semen, motile spermatozoa swim up into the culture. The upper part of the layered medium is then carefully removed for further use ( Boomsma *et al* ., 2019 ). Although inexpensive and fast, swim-ups require a near to normal semen sample with motile sperm and is not suitable for all types of semen samples seen in the assisted reproduction setting.

DGC separates spermatozoa according to their density. Motile, morphologically normal spermatozoa is aspirated from the solution with the highest concentration of gradient after centrifugation ( WHO, 2021 ), followed by subsequent washes using additional centrifugation steps to obtain a pure fraction of motile sperm. DGC techniques are easier to standardize compared to swim-ups and the results are more consistent ( Boomsma *et al* ., 2019 ). DGC is also suitable for all types of ejaculated semen samples and is the preferred method for male factor infertility due to higher total motile sperm recovered ( Henkel & Schill, 2003 ; WHO, 2021 ). But, sperm selection using DGC raises concerns regarding the negative effect of repeated centrifugation steps. In total, DGC protocols sums up to 40 minutes of centrifugation. There is an association between high sperm DNA fragmentation and poor reproductive outcomes for both intrauterine insemination ( Cho & Agarwal, 2017 ) and ICSI ( Lourenço *et al* ., 2023 ). DGC has been shown to result in increased sperm DNA fragmentation compared to swim-up ( Muratori *et al* ., 2019 ).

Here, we present our clinical validation of a microfluidics sperm selection device called ZyMōt™ and our experiences of using microfluidics in clinical practice for standard IVF. ZyMōt™ is an FDA-approved, CE-marked, single-use chamber that prepares sperm for use in intrauterine insemination, in vitro fertilization and intracytoplasmic sperm injection. Semen is placed under a membrane by injecting a specified volume semen into an inlet port. Suitable media is placed on top of the membrane, and then the chamber is incubated for 30 minutes. Motile sperm swims through the membrane filter and is collected through an outlet port. This yields a high fraction of motile sperm, with reduced sperm DNA fragmentation ( Quinn *et al* ., 2018 ).

The clinical validation was done in several steps; first the ZyMōt™ chamber was evaluated and compared against current DGC protocol using a split semen approach. Thereafter, a small sibling-oocyte study was done for standard IVF comparing ZyMōt™ to DGC, with fertilization rate, utility rate and embryo development and quality as endpoints. Thereafter, the ZyMōt™ sperm selection device replaced DGC for standard IVF in clinical practice. Key Performance indicators (KPI) were followed closely to evaluate the performance of sperm selection using ZyMōt™ for standard IVF.

## MATERIAL AND METHODS

All work was carried out at the Reproductive Medicine Center at the University Hospital of Örebro, Sweden.

### Ethical considerations

The study was approved by the Swedish Ethical Review Authority, ID 023-01153-0. The study was in accordance with the 1975 Helsinki declaration, reviewed in 2013.

### Pre-validation testing and outcomes; split semen comparisons

ZyMōt™ sperm separation device (CooperSurgical, Trumbull, United States) was compared to DGC. Samples with >3 mL volume were selected for comparisons, n=25.

Each sample was analyzed twice and in parallel. One aliquot was prepared by placing 2 mL of semen on top of two layers of density gradient solution (PureSperm, Nidacon, Sweden), before centrifugation at 300g for 20 minutes. Supernatant was removed and pellet containing sperm were washed by adding 5 mL of PureSperm Wash (Nidacon, Sweden) followed by centrifugation at 500g for 5 min. The wash was repeated so that the pellet was washed twice. Finally, the supernatant was removed, and pellet re-suspended in fresh media.

The other aliquot was prepared using the ZyMōt™ 0.85 mL device following the manufacturer’s instructions. In short, 0.85 mL of the ejaculate was added to the device through the inlet port. 0.75 mL of media was placed on top of the membrane, and 50 µL media was used to prime the outlet port. The device was incubated at +37°C, 6% CO_2_ for 30 minutes. Thereafter, 0.5 mL of suspension containing motile sperm was drawn from the outlet port and collected in a centrifugation tube.

Using a Makler chamber, 5 µL of each sperm solution was analysed under microscope. Number of progressive motile sperm, non-progressive motile sperm and immotile sperm were counted, concentrations, total sperm count, and total motile sperm count was calculated. Sperm counts were done blinded in regard to sperm selection method.

### Clinical validation and outcomes; sibling oocytes

The effect of sperm selection on fertilization and embryo development was examined in a sibling oocyte comparison from 11 cycles with >6 oocytes and >3mL semen volume, totaling to 158 oocytes (all fresh, and autologous). Half of the retrieved oocytes were fertilized with sperm prepared using density gradient as described above (40 min total centrifugation time) and half of the retrieved oocytes were fertilized using sperm selected by ZyMōt™ (no centrifugation). Fertilization was done by gamete co-incubation overnight using 250 000 progressive motile sperm per well in G-IVF+ covered by OVOIL (Vitrolife, Sweden) in conventional incubator set at +37.2°C, 6% CO_2_). After over-night incubation, the oocytes were stripped and placed G-TL with OVOIL overlay in EmbryoScope+ (Vitrolife, Sweden) at +37°C, 6% CO_2_, 5% O_2_ for continued culture and evaluation up till six days.

Fertilization was assessed at +16-18 hours post insemination (HPI). Presence of two pronuclei (2PN) and two polar bodies (2PB) was categorized as normal, 0PN categorized as absence of fertilization and 1PN or >2PN as abnormal fertilization. Total fertilization rate was calculated as number of all types of fertilization divided by number of inseminated cumulus oocyte complex (COC). 2PN rate was calculated as 2PN oocytes divided by inseminated mature oocytes, M2 oocytes, capable of fertilization. M2 was determined using obtained time lapse images and defined as oocytes with polar bodies as opposed to oocytes displaying geminal vesicle (GV oocytes) or oocytes absent of polar body (M1 oocytes).

Embryo development stage and quality was assessed according to the clinic standard operation procedures. Morphological quality was assessed by Gardner Schoolcraft criteria for expansion, inner cell mass and trophodectoderm at +114 and +140 HPI. Utility rate was calculated as the percentage of clinical useful embryos of 2PN oocytes, where a clinical useful embryo is at least 3BB on the Gardner Schoolcraft score qualifying for transfer and/or cryopreservation by vitrification.

Time lapse parameters were annotated by senior embryologists using the EmbryoViewer software version 7.8.1.2690. The following parameters were noted: PN number, fading of pronuclei (tPNf), timing of cell divisions (t2, t3, t4, t5, t6, t7, t8, t9+), time of compaction (tSC), time to morula (tM), time to blastulation (tSB), time to blastocyst (tB), time to expanded blastocyst (tEB), time to hatching (tHB). KIDscore D5 (version 3.2) was obtained from manual annotations in EmbryoViewer and iDAScore (version 1.2.0.0) were obtained from AI-based annotation of time lapse images from EmbryoScope+.

### Standard IVF using ZyMōt™ without centrifugation

After pre-clinical and clinical validation, ZyMōt™ replaced DGC for all standard IVF except for frozen thawed sperm samples and testicular sperms samples. Key performance indicators were used to assess performance. These included fertilization rate, utility rate and pregnancy outcomes. In total, standard IVF using ZyMōt™ without centrifugation was performed for 347 oocytes from 39 oocyte retrievals.

KPI for standard IVF using ZyMōt™ with centrifugation was compared to KPI based on historical DGC outcomes between 2017-2022 (time period selected as culture media and culture conditions have been the same as for 2023 and onwards).

### Standard IVF using ZyMōt™ with centrifugation

As KPI indicated deterioration in fertilization rates compared to historical DGC data, use of ZyMōt™ was halted while causes for poor performance was investigated. We discovered that new instructions for use from the manufacturer included a 5 min, 300g centrifugation step with subsequent media replacement after sperm separation prior to standard IVF. These steps were added to the sperm selection protocol and use of ZyMōt™ was resumed. Standard IVF using ZyMōt™ with a 5 min centrifugation step and media change was performed for 1850 oocytes from 191 oocyte retrievals.

### Statistical analysis

All the statistical analysis was performed using IBM SPSS Statistics Viewer (v 29.0). For sibling oocytes, laboratory outcomes such as maturity rate, 2PN rate, utility rate and semen parameters after processing were compared using Mann Whitney U-test. Time lapse parameters were compared using student´s t-test. Embryo quality scores were compared using Wilcoxon single rank test. For the unpaired study groups, baseline characteristics, laboratory outcomes and embryo quality score were compared using One-Way ANOVA, while maturity rate, fertilisation rates, utility rate and pregnancy rate were compared using Chi-2-tests. Throughout, *p* -values below .05 were considered significant.

## RESULTS

### Split semen comparisons

**Figure 1:**
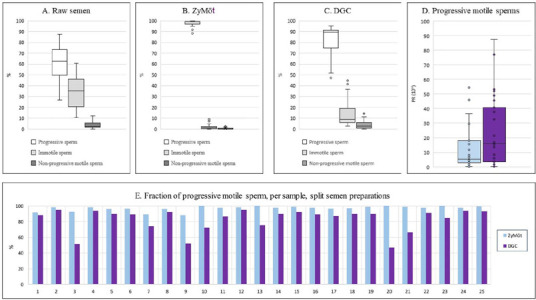
Split semen preparations. Semen samples, n=25, were prepared using ZyMōt™ (0.85mL) or density gradient centrifugation (DGC). A. Raw semen sample values, showing presence of progressive motile sperm, non-progressive motile sperm and immotile sperm. B. After sperm selection using ZyMōt™, enrichment of progressive sperms. C. Post sperm selection using DGC, enrichment of progressive sperm. D. Absolute number of progressive sperm after sperm selection for ZyMōt™ and DGC, higher number of progressive sperm after DGC. E. Per sample comparison of percentage of progressive sperm after sperm selection. Preparation with ZyMōt™ resulted in higher fraction of progressive sperms compared to DGC for all samples.

ZyMōt™ resulted in a higher fraction of progressive sperms compared to DGC (97.2±3.1% *vs.* 83.0±14.1%, *p* <0.01). In each sample, ZyMōt™ resulted in higher proportion of motile sperm compared to DGC, with just a few immotile sperms per sperm selection. DGC, however, resulted in almost double the number of progressive sperms retrieved after sperm selection (24.3±24.5x10^6^ *vs.* 12.5±14.9 x10^6^, *p* <0.01). See Figure 1D .

Importantly, using the clinic´s cut-off for deciding between standard IVF and ICSI, the same decision (standard IVF) would have been made in all but one sample, where ZyMōt™ sperm selection would have resulted in ICSI, but DGC would have resulted in standard IVF as fertilisation method.

### Clinical validation outcomes; sibling oocytes

The sibling oocytes showed no differences in fertilization rates, 77.4% (48/62) for ZyMōt™, and 77.6% (52/67) for DGC, *p* =.98). There was no difference in utility rate; ZyMōt™ 47.9% (23/48) *vs.* DGC 51.9% (27/52), *p* =.69. No cases of total failure of fertilization (TFF) defined as absence of 2PN oocytes was noted in the 11 cycles ( Table 1 ).

**Table 1:** Fertilization outcomes and embryo development outcomes on sibling oocytes, comparing ZyMōt™ to density gradient centrifugation (DGC). Clinically useful embryos refer to high-quality blastocysts, ≥3BB, transferred fresh or vitrified for later. KID-score and iDA score are expressed as median and range. The time lapse parameters are expressed as mean and standard deviation, hours post insemination (HPI).

Parameter	ZyMōt™	DGC	*p* -value
Retrieved ocytes (n)	69	74	-
M2 oocytes (n)	62	67	-
Maturity rate (%)	89.9	90.5	.89
2PN (n)	48	52	-
2PN rate (%)	77.4	77.6	.98
**Semen parameters (after processing)**			
Progressive motile sperms (%)	97.2±0.8	88.4±7.4	.002
Progressive motile sperms (millions)	31.7	17.6	.102
**Embryo development parameters**			
Clinically useful embryos (n)	23	27	-
Utility rate (%)	47.9	51.9	.69
KID-Score D5 (day 5 only)	7.1 (2.9-9-7)	7.3 (5.3-9.4)	.77
iDAScore (day 5 only)	8.7 (3.3-9.7)	8.2 (4.4-9.7)	.82
Time lapse parameters*			
tPNf	24.5±3.8	23.6±3.8	0.296
t2	27.1±3.5	27.1±4.6	0.966
t3	38.5±5.8	37.0±7.5	0.312
t4	41.8±7.6	41.7±12.0	0.944
t5	52.5±7.6	51.4±9.5	0.604
t6	54.3±8.2	54.2±7.2	0.947
t7	57.0±9.8	57.9±9.3	0.699
t8	60.4±11.5	60.4±9.0	0.987
t9+	73.7±8.2	73.2±7.0	0.813
tSC	80.1±6.5	81.5±7.6	0.537
tM	88.0±7.2	86.3±7.8	0.400
tSB	98.2±7.6	95.9±6.4	0.225
tB	108.7±12.7	105.7±8.5	0.324
tEB	112.3±7.7	113.2±7.2	0.710
tHB	114.8±9.9	118.6±9.9	0.362

PN=pronuclei, tPNf=time of PN fading, tSC=time of starting compaction, tM=time to morula, tSB=time of starting blastulation, tB=time to blastocyst, tEB=time to expanded blastocyst, tHB=time to hatching blastocyst. *= number of observations vary between parameters, missing data for later stages due to arresting embryos.

There were no differences in time lapse parameters or embryo quality (KID-score D5 and/or iDA score for day 5 blastocysts) ( Table 1 ).

### KPIs

Between 2017-2022, standard IVF using DGC on 8038 oocytes resulted in 4865 2PN oocytes (66.4% 2PN rate) and 2271 clinically useful embryos (46.7% utility rate), and transfer of 1338 embryos, the absolute majority as elective single embryo transfer - resulted in 646 positive HCG test (48.3% pregnancy rate). TFF was observed in 56 of 856 oocyte retrievals, corresponding to a TFF rate of 6.5% for standard IVF. See Table 2 for details.

**Table 2:** Baseline characteristics and Key Performance Indicators for standard IVF for all study groups. Presented for sperm prepared with density gradient centrifugation (DGC) 2017-2022, ZyMōt™ without centrifugation (Jan-Mar 2023) and ZyMōt™ with centrifugation (April 2023-May 2024). Age is presented as mean and standard deviation.

Parameter	DGC	ZyMōt w/o centrifugation	ZyMōt with centrifugation	*p* -value
Time period	2017-2022	2023, Jan-Mar	2023, April – 2024, May	
Patients	526	39	172	-
Cycle number information	856	39	191	-
1^st^ cycle (%)	80	77.3	75.6	n.s.
2^nd^ cycle (%)	15.8	13.6	14.0	n.s.
3^rd^ cycle or more (%)	4.2	9.1	10.5	<.05
**Baseline characteristics**				
Maternal age (years)	33.1±4.4	33.3±4.4	33.3±3.9	n.s.
Paternal age (years)	35.3±5.8	35.3±4.9	35.1±4.7	n.s.
Female infertility (%)	46.0	47.1	38.7	n.s.
Male infertility (%)	0.7	2.9	4.2	n.s.
Unexplained (%)	53.2	50.0	57.1	n.s.
**Laboratory outcome**				
COC (no)	8038	347	1850	-
M2 (n)	7324	293	1711	-
Maturity rate (%)	91.1	84.4	92.5	<.05
2PN	4865	174	1195	-
2PN fertilization rate	66.4	59.4	70.0	<.05
TFF, cycles (n)	56	6	13	-
TFF rate (%)	6.5	12.8	6.8	n.s.
Clinically useful embryos (n)	2271	66	480	-
Utility rate (%)	46.7	37.9	40.7	<.05
**Clinical outcome**				
Transferred embryos (n)	1338	50	218	-
Positive HCGs (n)	646	21	104	-
Positive HCGs (%)	48.3	42.0	47.8	n.s.

*COC=cumulus-oocyte complex, PN=pronuclei, TFF=total failure of fertilization, n.s=non-significant.*

### Standard IVF using ZyMōt™ without centrifugation

After introducing ZyMōt™ – without centrifugation -into clinical practice for standard IVF, the fertilization rate dropped to 59.4% (174/293) and the utility rate was 37.9 (66/174). These values were significantly lower compared to KPIs for standard IVF using DGC. Transfer of 50 embryos resulted in 21 pregnancies (42.0%), non-significantly different compared to KPIs for DGC. See Table 2 for details.

Importantly, without centrifugation and media change, 5 of 39 oocyte retrievals resulted in unexpected TFF, corresponding to 12.8 %. Although not statistically significant, the TFF rate for ZyMōt™ without centrifugation was twice as high as the TFF rate for DGC-fertilized oocytes between 2017-2022, *p* =.10.

### Standard IVF using ZyMōt™ with centrifugation

Protocol change – adding centrifugation with media change – increased the fertilization rate to 70.0% (1195/1711) which is significantly higher than ZyMōt™ without centrifugation, and similar with KPIs for DGC. Utility rate was 40.7% (480/1195) and TFF rate was 6.8% (13/191), which is almost identical to KPIs for DGC, and half of the noted frequency obtained for ZyMōt™ without centrifugation. Up until now, pregnancy rate is 47.8% (104 positive HCG test of 218 elective single embryo transfers) ( Table 2 ).

## DISCUSSION

This is, to our knowledge, the first study to evaluate ZyMōt™ for standard IVF in a clinical unselected setting. In the pre-clinical validation consisting of split sperm selection, sperm selection with ZyMōt™ resulted in lower number of progressive sperm available after preparation, but purer sperm selection with higher fraction of progressive sperm and with almost no immotile sperm in any of the samples. Consistently, using ZyMōt™ resulted in >95% progressive sperms. Purer preparations may be important as immotile/dead sperm might impact fertilization and/ or subsequent embryo development as they release ROS into the culture media during gamete co-incubation. Despite yielding less progressive sperm, sufficient number of sperms were retrieved with the 0.85 mL ZyMōt™ device to allow for standard IVF in all but one examined sample.

Sibling oocytes where half the oocytes were fertilized with sperms processed with DGC and half the oocytes were fertilized with sperms processed with ZyMōt™ resulted in the same fertilization rates and utility rate, and with no differences in embryo development speed or quality. Despite the positive outcome of the pre-clinical validation, when moving from DGC to ZyMōt™ for all standard IVF cases, fertilization rate was affected. There are a number of possible explanations for this. 1) In the sibling study, cases with more than 6 oocytes and semen samples with > 3 mL volume were included. These cases may not reflect the true clinical setting 2) the sample size for the sibling study was underpowered, possibly disguising true differences between the sperm selection methods 3) the majority of cycles resulted in fertilization rates as expected, but the number of unexpected TFF was increased (6/56, 12.8%) compared to this clinic´s KPIs (~6%). The cases with TFF significantly reduced the overall fertilization rate; when excluding the TFF cases, the 2PN fertilization rate was 64.2% which is acceptable, although not great. A TFF rate of 12.8 % is above the suggested threshold of <5 % suggested by ESHRE Special Interest Group of Embryology and Alpha Scientists in Reproductive Medicine ( ESIG/ALPHA, 2017 ) and indicates a definitive problem with achieving fertilization.

Of the six cases of TFF four cases were first time oocyte retrievals. The first treatment cycle has the highest risk of obtaining low fertilization of failure of fertilization with standard IVF. The risk has been estimated to 5-10 % in IVF ( Kahyaoglu *et al* ., 2014 ), so a few cases were expected. However, two couples with TFF using ZyMōt™ had previously done IVF in our clinic prior to the protocol change. TFF can be attributed to problem with sperm function, too few motile spermatozoa during insemination, or failure of oocyte activation ( Ebner *et al* ., 2015 ; Campos *et al* ., 2023 ). The number of motile sperm during insemination is controlled by ocular inspection by the embryologist, and hence too few sperms in the fertilisation well is unlikely. We reason that the most likely explanation for increased rates of TFF using ZyMōt™ without centrifugation, is insufficient removal of seminal plasma. Seminal plasma is a complex fluid secreted from several organs from the male genital tract. At the moment of ejaculation, sperms mix with the seminal fluids.

Thereafter, fertile sperms are separated from immotile sperms, debris and seminal plasma by active migration in the female genital tract. *In vivo* , the contact between sperms and seminal plasma is short but during this process progressively motile sperms are selected and undergo capacitation, which is a fundamental prerequisite for the sperm’s functional competence with regard to acrosome reaction ( Druart & de Graaf, 2018 ). Seminal fluid regulates capacitation, survival time in the female reproductive tract and conditions the female immune system. Importantly, seminal plasma factors regulates sperm capacitation ( Szczykutowicz *et al* ., 2019 ). It has been shown that TFF can be attributed to defective sperm decondensation ( Esterhuizen *et al* ., 2002 ) and so seminal plasma present in the final sperm solution may affect sperm decondensation and hence fertilisation. This was also the suggested explanation given by the sales representative.

The addition of a 5 min centrifugation with subsequent media change prior to gamete co-incubation aims at removing seminal plasma from the motile sperm fraction. Adding five minutes centrifugation and changing media restored fertilisation rates and TFF rates to normal. We therefore conclude that ZyMōt™ is suitable for gamete co-incubation overnight *only* when using the 5 min centrifugation step after sperm selection. When skipping the centrifugation step, there is an increased risk of fertilization failure.

Embryo development in terms of morphokinetics did not differ between sibling oocytes, indicating that fertilized oocytes developed in the same pace and sibling blastocysts obtained the same quality in terms of KID scores and/or iDAScore ( Table 1 ) regardless of being fertilized with sperms selected by microfluidics or DGC. Studies comparing embryo development between DGC and microfluidics in general are conflicting ( Yalcinkaya Kalyan *et al* ., 2019 ; Guler *et al* ., 2021 ). For ZyMōt™ specifically, several publications on ICSI have shown that ZyMōt™ selects sperm with less DNA damage ( Pardiñas Garcia *et al* ., 2022 ), that fertilization and blastulation are similar to DGC ( Tsuji *et al* ., 2022 ), and that following ICSI ZyMōt™ produces embryos with similar quality ( Quinn *et al* ., 2018 ). Our sibling oocytes were too few in number to draw firm conclusions on embryo development and quality, but future work might benefit from using AI-based time lapse data to capture any small differences in development capacity.

We add to the line of evidence on microfluidics that ZyMōt™ can also be used for standard IVF. Our work represents the clinical reality of a public/private clinic that performs IVF and ICSI based on semen quality and/or outcomes of previous treatment cycles. Approximately half the cases in our clinic are standard IVF, and the other half ICSI. Moving from DGC to microfluidics was driven by the desire to avoid centrifugation and hence improve sperm quality. Although centrifugation could not be completely avoided, the centrifugation time was reduced from 40 minutes to 5 for standard IVF. For ICSI, no centrifugation is needed after ZyMōt™, and for all ICSI samples at our clinic centrifugation is now avoided completely.

ZyMōt™ device is not appropriate for all samples. As it is based on microfluidics, motility is required. Therefore, additional sperm selection methods are needed in the clinic apart from the ZyMōt™ sperm devices. This is in line with the ESHRE guideline which states that sperm selection methods should be selected based on the properties of the sperm sample ( ESHRE Guideline Group on Good Practice in IVF Labs, 2016 ). Currently, the ZyMōt™ 0.85 mL is the standard protocol for all treatment types, but for low motility samples, severe oligozoospermia, frozen samples, testicular sperm samples DGC is used instead, and for low concentration but high-volume semen samples the 3 mL ZyMōt™ device is used instead when the treatment plan is standard-IVF.

Additional benefits of the ZyMōt™ device include time savings (in regards to hands-on time), the simplicity to adopt to the protocol, easy training and above all a short chain of custody with fewer movements between collection cups, centrifugation tubes and final prep tubes. This minimizes mismatching risks, especially in clinics that do not require double witnessing.

In conclusion, the microfluidic based ZyMōt™ sperm selection device has replaced density gradient centrifugation for both ICSI and IVF. This study supports the use of ZyMōt™ for standard IVF only with a five-minute centrifugation and media change prior to gamete co-incubation. This results in key performance indicators above accepted thresholds for the clinic.
